# A method to study the effect of bronchodilators on smoke retention in COPD patients: study protocol for a randomized controlled trial

**DOI:** 10.1186/1745-6215-12-37

**Published:** 2011-02-10

**Authors:** WD van Dijk, PTJ Scheepers, R Cremers, JWM Lenders, W Klerx, C van Weel, TRJ Schermer, Y Heijdra

**Affiliations:** 1 Department of Primary and Community Care, Radboud University Nijmegen Medical Centre; 2 Department of Epidemiology, Biostatistics and HTA, Radboud University Nijmegen Medical Centre; 3 Department of Internal Medicine, Radboud University Nijmegen Medical Centre, Nijmegen, the Netherlands; 4 Department of Internal Medicine III, University Hospital Carl Gustav Carus, Dresden, Germany; 5 Food and Consumer Product Safety Authority, Eindhoven, the Netherlands; 6 Department of Pulmonary Diseases, Radboud University Nijmegen Medical Centre, Nijmegen, the Netherlands

## Abstract

**Background:**

Chronic obstructive pulmonary disease (COPD) is a common disease, associated with cardiovascular disease. Many patients use (long-acting) bronchodilators, whilst they continue smoking alongside. We hypothesised an interaction between bronchodilators and smoking that enhances smoke exposure, and hence cardiovascular disease. In this paper, we report our study protocol that explores the fundamental interaction, i.e. smoke retention.

**Method:**

The design consists of a double-blinded, placebo-controlled, randomised crossover trial, in which 40 COPD patients smoke cigarettes during both undilated and maximal bronchodilated conditions. Our primary outcome is the retention of cigarette smoke, expressed as tar and nicotine weight. The inhaled tar weights are calculated from the correlated extracted nicotine weights in cigarette filters, whereas the exhaled weights are collected on Cambridge filters. We established the inhaled weight calculations by a pilot study, that included paired measurements from several smoking regimes. Our study protocol is approved by the local accredited medical review ethics committee.

**Discussion:**

Our study is currently in progress. The pilot study revealed valid equations for inhaled tar and nicotine, with an R^2 ^of 0.82 and 0.74 (p < 0.01), respectively. We developed a method to study pulmonary smoke retentions in COPD patients under the influence of bronchodilation which may affect smoking-related disease. This trial will provide fundamental knowledge about the (cardiovascular) safety of bronchodilators in patients with COPD who persist in their habit of cigarette smoking.

**Trial registration:**

ClinicalTrials.gov: NCT00981851

## Background

Chronic obstructive pulmonary disease (COPD) is a common chronic disease, characterised by poorly reversible and progressive airflow obstruction. A substantial fraction of COPD-related mortality and morbidity is due to cardiovascular disease [1-3]. Both diseases share an important risk factor: smoking. Cigarette smoking causes over 80% of COPD, whereas 20% of cardiovascular mortality is attributable to smoking [4,3,6]. Cessation of cigarette smoking is an important prognostic factor in patients with COPD and cardiovascular disease. Besides, smoking cessation is essential in preventing development of cardiovascular disease in all people, including COPD patients. However, half of COPD patients are persistent smokers [7,4,9,5].

Meanwhile, the cornerstone of pharmaceutical treatment of COPD symptoms is bronchodilation. It seems rational however, that bronchodilation diminishes the hyperinflated state of the lung, enhances deeper smoke inhalation and as a result increases the pulmonary deposition or uptake of pathogenic cigarette smoke constituents. Concurrently, the amount of cigarettes smoked - i.e. the amount of smoke - is related positively to cardiac mortality [4,5,10]. Therefore, we hypothesised a hazardous interaction between chronic bronchodilation and smoking, that would result in increased cardiovascular morbidity and mortality in COPD patients who persevere in smoking [11]. If the hypothesized interaction does indeed exist, the consequences in terms of pharmacotherapeutic management of patients with COPD would be substantial. However, no strong evidence to substantiate our hypothesis has been published yet. With the randomised controlled trial described in this paper we aim to expose the fundamental mechanism of interaction. Similar studies on smoke retention in general have been reported, but not in relation to bronchodilator treatment or in patients with COPD [12-15].

Our first objective is essentially to demonstrate a proof of concept of the fundamental underlying assumption: does administration of a bronchodilator to a patient with COPD lead to increased retention of cigarette smoke constituents. In this article we describe the design and methods of our study that determines the effect of bronchodilation on smoke retention in COPD patients when they smoke a cigarette. In addition, we determine the effect of bronchodilation on smoking patterns and short-term biological effects associated with cardiovascular disease. Apart from our methods, we report the results of our pilot study that validated the retention measurements.

## Method

Interaction in COPD experiment (ICE) is a randomised trial designed to evaluate the effect of bronchodilators on smoke retention in COPD patients.

### Study design

The study is designed as a double-blinded, placebo-controlled, randomised crossover trial, in which 40 COPD patients - who are current smokers - smoke a cigarette under controlled conditions, both during undilated and maximal bronchodilated conditions during two separate sessions. Participants have a 'wash-out' period of 1 week between both sessions and the experiment always initiates between 8:00 and 9:00 AM. The study is registered at http://www.clinicaltrials.gov identification number NCT00981851.

### Interventions

After inclusion, patients are randomly allocated to commence with one of both bronchodilated conditions. During one session, participants smoke a first cigarette, have their medication administered directly after, and 45 minutes later they smoke their second cigarette.

• ***Bronchodilation***. To accomplish both bronchodilated conditions, patients receive either a combination of potent bronchodilating drugs - 5 μg tiotropiumbromide dry powder inhalation by Respimat as well as 400 μg aerosolized salbutamol by volume spacer - or a combination of their placebos. These bronchodilators ensure a gradual and maximal bronchodilation after 45 minutes and both offer similar placebos [16,17]. In order to initiate the experiment undilated, patients have to refrain from bronchodilators according to table 1. Patients are allowed short-acting bronchodilators up to 8 hours before the experiment to minimise bronchodilator withdrawal. To minimise intra-individual variation in baseline bronchoconstriction, patients should not have had an exacerbation or have used more than usual short-acting rescue medication within the previous week.

**Table 1:** Duration of bronchodilator abstinence

Abstinence (hours)	Bronchodilator
• 8	• *Salbutamol* • *Terbutaline* • *Ipratropium bromide*
• 24	• *Formoterol* • *Salmeterol*
• 48	• *Tiotropium bromide*

• ***Smoking***. Smoking occurs according to well controlled protocols. To neutralise the effect on smoking patterns by nicotine craving, tobacco smoking is accepted up to 8 hours before the experiment and patients have a cigarette prior to medication administration. During all study sessions patients smoke CM6 cigarettes, a non-commercial cigarette with relatively little variation in smoke yields [18]. Cigarettes are conditioned at 22°C and at 60% relative humidity - according to ISO standards - in an incubator with a saturated mixed salt solution [19]. Cigarettes are ignited electrically. Participants wear a nose-clip, and are instructed to inhale all smoke, exhale into a mouthpiece connected to Cambridge filters and to smoke the cigarettes up to a marking spot 32 mm from the tipping end. Cigarettes are extinguished by pulling off the burning core to preserve the cigarette filter.

The interventions are executed at a laboratory location, separated from patient care, specifically facilitated to avoid smoke exposure of researchers and department personnel. The local authorised university safety-board and housing-board approved this facility in accordance to national and local smoking legislation.

### Eligibility

Patients are selectively recruited as from October 2009 by their own respiratory consultant or respiratory nurse during regular scheduled clinic visits, at the pulmonary diseases department of the Radboud University Nijmegen Medical Centre. Eligible candidates are initially selected from pulmonary patient files, based on the selection criteria (table 2). We recruit patients with COPD (defined according to current GOLD criteria), [3] without interfering co-morbidities, who are current smokers, and who are capable of fulfilling the experiment physically and logistically. Patients with interfering factors such as asthmatic features or other interfering non-COPD respiratory disorders are excluded.

**Table 2:** Eligibility

Inclusion criteria	Exclusion criteria
• COPD GOLD stage II-III (i.e. FEV_1_/FVC <0.70 and FEV_1 _30-80% of predicted value). • Current cigarette smoking. • Willing to provide written informed consent. • Willing to refrain from smoking and avoid use of a bronchodilator >8 hours. • Registered in one of the recruitment institutes.	• COPD GOLD stage I or IV. • Active asthmatic component: present asthma by complaints, positive histamine provocation test, eosinofilia or reversibility ≥ 10% of predicted. • Unable to perform the whole experiment physically or due to communication problems. • Recent, active and relevant non-COPD respiratory disorders.

Candidates receive written information and have at least one week to consider their participation. The investigator (WvD) contacts candidates by telephone to address further questions and to provide additional information if necessary. Participants sign an informed consent form. The encoded identities are only accessible by the investigator and research assistant. The local accredited medical review ethics committee approved our protocol: CMO region Arnhem-Nijmegen, CMO 2009/037. We explicitly support the quit-smoking advice and do not interfere if a patient has initiated an attempt to quit smoking.

### Blinding

Placebo and bronchodilator administrations are double-blinded. Blinding is only known to a research nurse, who prepares and delivers the medication for each study participant, and does not have any other involvement. The placebos are not distinguishable from the bronchodilating drugs through appearance or administration. Pulmonary function tests after drug administration are not performed until the final cigarette has been smoked. We will unblind all study medication simultaneously after the final participant has concluded the experiment or individually in case of a serious adverse event.

### Sample size calculation

**Figure 1:**
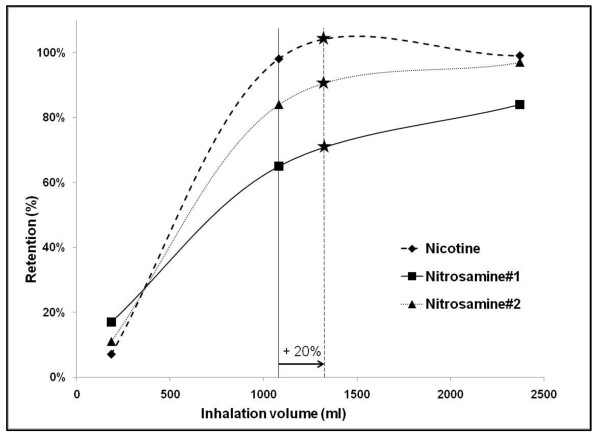
20% increase (---) of a normal inhalation volume (--) leads to a mean 5% increase in retention (*) of 3 main tar compounds.

In a cross-over design with paired samples, a number of 34 patients is sufficient to demonstrate a medium standardised effect size (δ = 0.5) of pre and post mean difference between the two different conditions (assumptions: α = 0.05, 1-β = 0.80, two-tailed testing, c = 7.9): n = c/δ^2 ^+ 2 [20]. The medium standardised effect size is derived from an interpretation of Feng's study about retention dynamics [13]. We base the increase in tar retention on the assumption that our participants execute an average smoking pattern, that bronchodilation results in a 20% increase of Forced Vital Capacitiy (FVC), and that this increase results in 20% more as well as deeper smoke inhalation [9]. Figure 1 shows our interpretation of Feng's study and reveals a subsequent estimated 5% increase in tar retention caused by bronchodilation. The mean standard deviation of tar retention approximates 10%, based on three main tar substances, and results in an effect size of 0.5 (increase/standard deviation) [13].

### Outcome measures

Primary outcome will be the evaluation for both bronchodilated conditions of changes in cigarette smoke retention between before and after medication administration. These retentions are calculated by both the inhaled tar and nicotine weights (mg) minus their exhaled amount, divided by the inhaled amount [13].

Secondary outcomes include smoking patterns - presented by mean inhalation as well as exhalation volume (ml) and time (seconds) - and respiratory function by Forced Expiratory Volume in the first second (FEV_1_) and FVC. These variables will also be analysed for their correlation with smoke retention. Another secondary outcome is the short-term reaction of biomarkers associated with cardiovascular disease in long-term studies, i.e. C-reactive protein and platelet activation by fibrinogen [21-23]. Study outcomes are presented in table 3.

**Table 3:** Study outcomes

Outcome	Variable
**Primary outcome**	**Smoke retention**
	• *Tar (%)* • *Nicotine (%)*
**Secondary outcomes**	**Pulmonary function**
	• *FEV*_*1 *_*reversibility (%)* • *FVC reversibility (%)*
	**Smoking pattern**
	• *Mean inhalation time and volume (sec + ml)* • *Mean exhalation time and volume (sec + ml)* • *Total smoking time (min)* • *Amount of puffs (n)*
	**Biomarkers**
	• *CRP and high sensitivity CRP (mg/l)* • *Fibrinogen (mg/l)*

### Measurements

#### Pilot study

Tar and nicotine yields are related to the amount of nicotine in cigarette filters [13,15]. We performed a pilot study at the Food and Consumer Product Safety Authority, Eindhoven, the Netherlands (FCA) to determine these relations for CM6 cigarettes, when artificially smoked up to 32 mm from the tipping end. A Cerulean SM450 smoking machine performed smoking at 6 different smoking regimes - ranging from 35 ml to 55 ml per 2-second puffs, at 1 and 2 puffs per minute. After smoking, whole cigarette filters were extracted in a 20 ml isopropanol-based solution and analysed for their nicotine amounts by gas chromatography, according to standardised operating procedures (ISO method 10315). For single cigarettes, tar was captured on a paired Cambridge filter pad, which was weighed before and after smoking to generate a gross tar weight. Subsequently these filters were extracted and analysed similar to the cigarette filters for their nicotine amount - i.e. nicotine yield - and water amounts. Subtracting the water amount from the gross tar weight, corrected by addition of the water amount of blank filter pads, resulted in the net tar weight, i.e. tar yield (ISO method 4387). The paired values resulted in an equation to calculate inhaled nicotine and tar amounts by cigarette filter nicotine weights.

**Figure 2:**
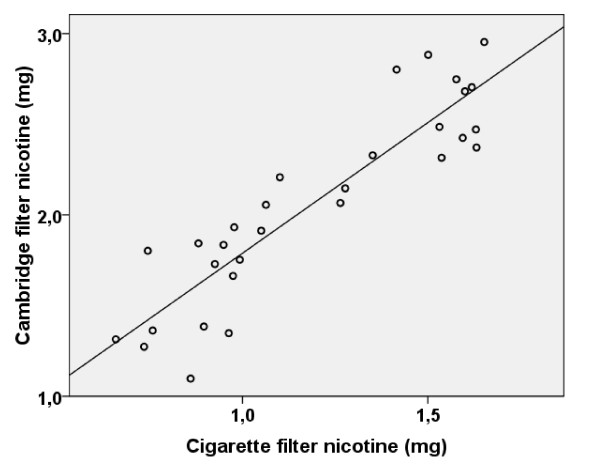
**Correlation between cigarette and Cambridge filter nicotine (Pearson's correlation coefficient of 0.90)**.

From our pilot study, we obtained 30 valid results of paired CM6 cigarette filters and Cambridge filters, by 6 different smoking regimes, and generated two equations to estimate the inhaled amounts of tar and nicotine for our participants: nicotine inhalation (mg) = 1.4 * nicotine (cigarette filter) + 0.35 (R^2 ^= 0.82, p < 0.01, figure 2). Tar inhalation (mg) = 20.8 * nicotine (cigarette filter) + 1.06 (R^2 ^= 0.74, p < 0.01).

#### Baseline assessment

By questionnaire we attain medical history, smoking history, all medication use, and - prior to both sessions - recent use of cigarettes, bronchodilation and rescue medication. FEV_1 _and FVC are measured each session by a portable spirometer - Micro loop 36-ML3535MK8, Carefusion. A comprehensive baseline pulmonary function test is by spirometry: Total Lung Capacity (TLC), Diffusion and Inspiratory Vital Capacity (IVC). Participants wear an inductive plethysmography garment - Vivometrics Lifeshirt^® ^- that measures single smoking puffs real-time and facilitates smoking pattern analyses by Vivologic software [13]. We calibrate the Lifeshirt^® ^by 10 simultaneous spirometric mean tidal volume measurements. We attain baseline biomarkers by venous blood samples. Table 4 summarises all baseline characteristics.

**Table 4:** Baseline characteristics

Variables	
**History** • *COPD GOLD-classification* • *Pulmonary and other diseases* • *Smoking*	**Medication** • *Pulmonary medication* • *Co-medication*
**Pulmonary function** • *FEV*_*1 *_*pre- and post bronchodilation* • *FVC pre- and post bronchodilation* • *Inspiratory Vital Capacity* • *Total Lung Capacity* • *Diffusion*	**Pre-experiment condition check** • *Last smoke* • *Last bronchodilator use* • *Rescue bronchodilator last week?* • *Exacerbation last week?*

#### Experiment

Each session, participants smoke two cigarettes while we mark smoke inhalations manually for the Lifeshirt^®^. In addition, these puffs will be visually identified during the Lifeshirt data management. After smoking the second cigarette, we measure FEV_1 _and FVC twice by handheld spirometry to determine both airflow obstruction and reversibility. We repeat blood sampling from the same vein twice as well. In order to establish inhaled tar and nicotine weights, we analyse the cigarette filters at the FCA for their nicotine contents. We establish exhaled tar and nicotine weights by analyses of Cambridge filters similar to the pilot study [12,13]. These Cambridge filters capture approximately all tar. To diminish losses, participants wear a nose clip, are instructed to inhale all smoke, and exhale all smoke through a mouthpiece, which connects to two parallel 55 mm Cambridge filters. We use inert PTFE (Teflon) tubing, PTFE filter pad holders and metal connectors, and maintained temperature at 40°C to prevent smoke sedimentation and condensation and to prevent filter pads from blocking. We reduced the dead space to 35 ml. Filter pads are re-weighed after smoking, only after water evaporation has stabilised. All filters are vacuum sealed and preserved at room temperature. Prior to analyses at the FCA, filters are re-stabilised at 22°C at 60% relative humidity [13,14].

### Statistical analysis

We use SPSS 16.0 to statistically analyse our results. We compare medication induced changes in cigarette smoke retention between the placebos and bronchodilators, by mixed model analyses. Baseline characteristics are studied as determinants of short-term changes in smoke retention and cardiovascular biomarkers by univariate analysis of variance (ANOVA). Pulmonary function and smoking pattern are studied for a correlation with smoke retention by a separate correlation analyses. Regression analyses was used for our pilot study.

## Discussion

The aim of our study is to demonstrate a fundamental effect of bronchodilation on smoke retention as a proof of concept for our hypothesis that suggests a hazardous interactive effect of bronchodilation and smoking on cardiovascular disease in COPD patients. The study design is directed at selecting a population in which the experiment is rather safe and where we expect some bronchodilator effect on the FVC: COPD Gold classification II-III. In addition, this group represents 70% of COPD patients. We therefore believe, selection bias is minimal and generalisability is reasonable [24].

The power of our study benefits from the crossover design which reduces the required sample size. The study effect is amplified by maximum bronchodilation in one session - by combining a beta-2 agonist and anticholinergic bronchodilator - and maintaining maximum deprivation of bronchodilation in the other session. Validity of the study is consolidated by the following: participants have a break of one week between the first and second measurement session to eliminate any carry-over effects. Sessions are scheduled at standardised times to decrease periodicity by cyclic daytime influences [25]. Randomisation and blinding throughout the experiment is guaranteed by independent research assistants, undistinguishable placebos and post medication pulmonary function tests after the second cigarette only. The way patients for the study are recruited and all other procedures used in the study are conform ethical standards. Participants do not have to smoke or take medication differently than they are used to except for pre-experimental abstinence. We optimised exhaled tar and nicotine measurements by heating of the construction to exclude smoke condensation, inert materials and a small dead space to avoid sedimentation, and clear instructions and a nose clip to prevent smoke losses.

We measure inhalation and exhalation tar and nicotine amounts by a method similar to methods that have been proven valid in previous studies [12-15]. To minimise variation in inhaled amounts of tar and nicotine, we standardised the measurements by conditioning of the cigarettes, lighting them electrically, and smoking them up to 32 mm from the tipping end. In addition, by utilising just one cigarette brand, we are able to exclude the variation of the brand-specific nicotine filtration efficiency. A relatively suboptimal correlation between cigarette filter nicotine and inhalation amounts is likely due to the influence of different smoking regimes, specifically puff volume. Still, we assume our crossover-based method is valid since differences in smoking regimes consist merely between patients and not as much within patients. Furthermore, daily differences in smoke patterns are accounted for by similar nicotine deprivation for both conditions and retention differences between before and after medication both sessions.

## Conclusion

We believe we developed a valid method to study the fundamental interaction between bronchodilation and cigarette smoking in COPD patients that may result in different pulmonary smoke retentions and parallel short-term cardiovascular effects. The pilot study on individual smoke yield measurements appears useful for future smoke studies. Our method reduces sample size and the individual burden by participation is limited. We expect that our study will provide crucial new insights on the safety of prescribing bronchodilators to patients with COPD who persist in their habit of cigarette smoking.

## List of abbreviations

ANOVA: Analysis of Variance; CM6: Coresta Mointor number 6; CMO: committee for human-related research; COPD: Chronic Obstructive Pulmonary Disease; FCA: Food and Consumer Product Safety Authority; FEV_1_: Forced Expirator Volume in 1 second; FVC: Forced Vital Capacity; GOLD: Global initiative for chronic Obstructive Lung Disease; ICE: Interaction in COPD Experiment; ISO: International Organisation for Standardisation; IVC: Inspiration Vital Capacity; TLC: Total Lung Capacity;

## Competing interests

None of the authors and none of the relatives of the authors has a relation to, or income from, the tobacco industry.

As government official from the Dutch Food and Consumer Product Safety Authority (VWA), Walther Klerx is involved in law enforcement of tobacco products, though independent of the tobacco industry. In addition, the VWA (and Walther Klerx as technical expert) is a member of the task force tobacco laboratories of the WHO and EU and therefore obliged to comply to article 5.3 of the FCTC (to prevent conflicts of interest with the tobacco industry of any kind). Chris van Weel received fees or grants as paid to a GOLD member and used for research, education, equipment, salaries, etc. <$10,000: Bayer, NovoNordisk. >$10,000: Astra Zeneca, Boehringer Ingelheim, GlaxoSmithKline, Novartis. Wouter van Dijk, Paul Scheepers, Robbert Cremers, Jaqcues Lenders, Tjard Schermer and Yvonne Heijdra do not have any conflict of interest.

## Authors' contributions

WvD developed the design and protocol, carried out the statistical analyses and drafted the manuscript. PS assisted in statistical analyses and developing the study protocol, particularly the smoke inhalation and exhalation. RC performed the experiments for smoke inhalation, participated in the statistical analyses and assisted in developing the protocol. JL assisted in developing the protocol, particularly the measurement of biomarkers. WK assisted in developing the protocol, particularly the inhalation and exhalation measurements. CvW participated in developing the study design. TS assisted in developing the design and protocol. YH assisted in developing the design and protocol, particularly the Lifeshirt measurements. All authors helped to draft the manuscript and approved the final version.
